# Comparison of classical and transgenic genetic sexing strains of Mediterranean fruit fly (Diptera: Tephritidae) for application of the sterile insect technique

**DOI:** 10.1371/journal.pone.0208880

**Published:** 2018-12-14

**Authors:** José S. Meza, Ihsan ul Haq, Marc J. B. Vreysen, Kostas Bourtzis, Georgios A. Kyritsis, Carlos Cáceres

**Affiliations:** 1 Insect Pest Control Laboratory, Joint FAO/IAEA Division of Nuclear Techniques in Food and Agriculture, International Atomic Energy Agency, Vienna, Austria; 2 Programa Moscafrut, SAGARPA-IICA, Metapa de Domínguez, Chiapas, México; 3 National Agricultural Research Centre, Islamabad, Pakistan; University of Thessaly School of Agricultural Sciences, GREECE

## Abstract

The development of genetic sexing strains (GSSs) based on classical genetic approaches has revolutionized the application of the sterile insect technique (SIT) against the Mediterranean fruit fly *Ceratitis capitata* (Wiedemann) (Diptera: Tephritidae). The global use of Mediterranean fruit fly GSS for SIT applications as part of area-wide integrated pest management (AW-IPM) programmes is testimony to their effectiveness. During recent years, transgenic sexing strains (TSSs) have been developed through genetic engineering techniques offering the possibility to produce male-only progeny by introducing female embryonic lethal genes and to increase the efficacy to identify released sterile males by means of the expression of fluorescent transgene markers. Here, we present a comparative analysis of two Mediterranean fruit fly strains: the classical GSS VIENNA 8^D53-^/Toliman and the transgenic FSEL#32. The strains were compared for production efficiency and quality control indices under semi mass-rearing conditions, response to sterilizing irradiation doses, male mating performance in walk-in field cages, and production cost of male-only pupae. The results showed that, the FSEL #32 TSS had a similar fecundity but a higher production of male-only pupae than the VIENNA 8^D53-^/Toliman GSS. For some of the quality control parameters tested, such as pupal weight and survival under starvation conditions, the FSEL #32 TSS was inferior to the VIENNA 8^D53-^/Toliman GSS. Both the transgenic and the classical genetic sexing strains have shown acceptable and similar mating competitiveness when compared with wild males for mating with wild females. The cost production for both strains is similar but the FSEL#32 TSS may potentially be more cost effective at higher production levels. The results are discussed in the context of incorporating the transgenic strain for SIT application.

## Introduction

The Mediterranean fruit fly *Ceratitis capitata* (Wiedemann) is one of the most destructive pests of fruits in the world. It causes economic losses by damaging the fruits and impeding international horticultural trade [1, 2]. Conventional control measures, relying on synthetic insecticides have been proven ineffective and have several disadvantages [3, 4], which has resulted in demands for alternative control tactics that are friendlier to the environment. During the last forty years, this pest has been successfully managed by incorporating the sterile insect technique (SIT) as an integral component of area-wide integrated pest management (AW-IPM) approaches [5]. The SIT relies on releasing mass-reared sterile males in the target area where they transfer their sterile sperm to wild females, which then cannot produce offspring. The successive releases of high quality sterile males in a systematic way can lead to suppression or eradication of a wild population [6]. The SIT integrated as a component of an AW-IPM program has major advantages, i.e. it is environment friendly, species specific, doesn’t add new genetic material in the target area, has very good benefit-cost ratios, is politically acceptable, and is perceived by the general public as something positive [7]. As only the sterile males are required to induce sterility in the wild population, the production, sterilization and release of both sexes cause additional cost, reduce the efficiency of the SIT application due to the assortative matings of the sterile females with the sterile males and may further reduce the quality of fruits due to the stings of released sterile females [8]. Therefore, efforts have been made to develop genetic sexing strains (GSS) that allow the removal of females from the production line and offer the opportunity for sterile male-only releases. The development of GSS that allowed only male releases and the ability to produce Mediterranean fruit flies on an industrial scale are the two main factors that contributed to the worldwide deployment of SIT applications against this pest [9].

The ‘first generation’ GSS of the Mediterranean fruit fly was constructed by classical genetic approaches in which the wild type allele of the white pupae gene (white color) was linked to the male determining region by means of a translocation between an autosome and the Y-chromosome. This enabled the separation of female white pupae from male brown pupae by optical and mechanical means [10] and consequently made sterile male-only releases possible. However, the costs of rearing both male and female larvae remained rather high, as the separation of the sexes was done at the pupal stage. Secondly, the separation of male and female pupae was not 100% accurate and often caused damage to the male pupae [11]. Therefore, a sexing system that could separate the sexes at an earlier developmental stage, e.g. during embryogenesis, would be more cost effective. Such a sexing system became reality with the development of a ‘second generation’ GSS that was based on a *temperature sensitive lethal* (*tsl*) mutation [12]. Female individuals were homozygous for the *tsl* mutation and hence, sensitive to elevated temperatures. Exposing eggs at 34°C for 12 h eliminates all females at the embryonic stage [13], and thus female rearing in a male-only production line could be avoided. Although no thorough comparative cost analysis was performed between the first and the second generation of GSS, the main advantage of the latter (*tsl*-based GSS) was the reduction in space and cost during the larval rearing process.

In general terms, the production costs of the *tsl*-based GSS was slightly higher than that of the bi-sexual strain due to the lower productivity of the *tsl*-based mutation [14], there were significant cost savings with respect to the holding of sterile males in the emergence and release facilities (saving of space as there was no need to hold the female flies) and the release of only males in the field (number of males released could be doubled per flight, due to the absence of females). Furthermore, male-only releases increased the biological efficiency of the SIT application at least 3-fold (e.g. no assortative mating) which accounted for enhanced cost effectiveness of SIT application [15, 16].

Despite the numerous advantages of GSSs, the first GSS developed had certain limitations such as the lack of genetic stability under mass-rearing conditions and the appearance of aberrant sex recombinants. In order to reduce the recombination frequency, several GSS were constructed that aimed at keeping the physical distance between the Y-5 autosome translocation breakpoint and the two closely linked selectable markers (*wp* and *tsl*) that are closely linked as short as possible [17, 18]. Thus, improved versions of the GSS such as VIENNA 7 had the Y-5 autosome translocation breakpoint (5R:58B) closer to the markers than in many other GSS [19], and as a result, showed 80% less recombination as compared with an earlier version of the GSS T(Y;5)101. To further increase the genetic stability of the GSS, a suppressor of crossing-over between the wild type alleles pseudo-linked to the “Y” chromosome and selectable genetic markers alleles was created by the induction of the D53 homozygous viable chromosomal inversion in the autosome 5, which covers the region from 5L:50B to 5R:59D. The D53 inversion was included into the T(Y;5)101 translocation line, which is 10–30% more productive than others, resulted in a new GSS, called VIENNA 8 or VIENNA 8^D53+^ (see chromosomal map in reference 13). However, whereas the larger Mediterranean fruit fly rearing facilities in Argentina, Guatemala, Mexico, Spain, and the USA still are using the GSS T(Y;5)101 without inversion (hereafter named VIENNA 8^D53-^), other facilities in Brazil, Chile, Israel, Peru, South Africa adopted the VIENNA 8^D53+^ and in Australia VIENNA 7, which doesn’t carry the D53 inversion [20].

In addition, the introduction of a filter rearing system (FRS) in mass-rearing facilities greatly assisted in mitigating the problem of the persistent breakdown of the strain due to male genetic recombination and facilitated the maintenance of the genetic integrity of the strain. In an FRS, a small “mother” colony is maintained under relaxed rearing conditions that provide flies for colony amplification. All flies that retrofit the mother colony are screened, and all aberrant males and females are removed to insure the integrity of the GSS. Colony amplification is done typically for 2–3 generations until adequate numbers of male insects are produced for field releases. The main characteristic of a FRS is that none of the flies produced in the amplification line is returned to the mother colony [21].

In addition to the GSS developed using classical Mendelian genetics, several efforts have been undertaken to develop “transgenic sexing strains” (TSS) through genetic engineering [22]. One of the approaches was based on a tetracycline-regulated system that produces a synthetic tetracycline trans-activator (tTA). In the absence of tetracycline the lethal gene is expressed, and in the presence of tetracycline (added in the water at the adult stage) the tTA is blocked, thus preventing the expression of the lethal gene (Tet-off system) [23, 24]. The incorporation of the Mediterranean fruit fly sex-determination gene *transformer* (*Cctra*) into the Tet-off system resulted in a strain with female-specific lethality controlled by the absence/presence of tetracycline [25, 26]. More recently, a female-specific embryonic lethality (FSEL) system was developed in the Mediterranean fruit fly by combining the female-specific tetracycline-repressible lethality with promoter/enhancers of cellularization genes to restrict the tTA expression and the pro-apoptotic gene *hid* that provoked specific female lethality at the embryonic stage [27].

In this study, a comparison was made between two strains that offer the possibility to induce female embryonic lethality and that were developed using classical genetics and transgenic approaches, i.e. the VIENNA 8^D53-^/Toliman GSS and the FSEL#32 TSS. An assessment was made of their production efficiency, quality control parameters under semi-mass rearing conditions, the effect of gamma irradiation on male mating competitiveness in walk-in field cages, and a comparative cost analysis. In addition, the quality control indices of the strains were compared in the modified sequential phases of production. Weakness and strengths of both sexing systems are discussed based on the outcomes of the experiments.

## Material and methods

### Study site

The study was carried out at the Insect Pest Control Laboratory (IPCL) of the Joint FAO/IAEA Division of Nuclear Techniques in Food and Agriculture, Seibersdorf, Austria (UTM zone 33N, Coordinates 613364, 5314206).

### Study insects

#### VIENNA 8^D53-^/Toliman

VIENNA 8^D53-^/Toliman is a GSS (described earlier) that has been reared at the “El Pino” and Metapa mass-rearing facilities in Guatemala and México [20], respectively for the production and release of sterile males for the SIT application.

#### FSEL#32

FSEL#32 is a TSS with a female specific embryonic lethality system and a binary expression system, i.e. one is the driver system that is marked with the DsRed gene, and the other is the sexing effector system marked with a green fluorescence EGFP gene, so the insects have red and green fluorescence expression [27]. Adding 10 μg tetracycline/mL of water for adult feeding (Tet+) the system allows the production of males and females, whereas all female embryos are eliminated if the water is tetracycline free (Tet-). The FSEL#32 TSS (FSEL#31 hereafter) was developed at the Institute für Zoologie und Entwicklungsbiologie, Georg-August-Universität Göttingen, Germany and provided to the IPCL to characterize the strain under semi-mass rearing conditions.

#### Wild strain

The wild flies were obtained from pupae collected in infested orchards of sour oranges (*Citrus x aurantium*, L) at Llombai (Ribera Alta, Valencia) Spain (UTM zone 30N, Coordinates 707544.91, 4352440.36). The flies were reared on citrus fruits to keep its “wildish” status as much as possible. Flies from the F_1_ generation were used for the experiments.

### Experimental design

Mass rearing of all strains studied was performed for 4 consecutive generations, thus obtaining four replicates. Pupae production and associated parameters were replicated three times per generation, obtaining in total 12 replicates for each production line (male-only and colony; both sexes) and sexing strain for the entire experiment. The experimental design of other tests (survival of males under starvation, genetic stability, induced sterility and mating competitiveness) and parameters assessed, which are not associated with production itself, is described below.

### Semi mass-rearing

The GSS and the TSS were reared for 4 consecutive generations under semi mass-rearing conditions, producing nearly two million pupae per generation, and evaluated as described below.

#### Colony

Adult flies were maintained in standard Mediterranean fruit fly mass-rearing cages that consist of an aluminium frame (180 x 180 x 20 cm) covered on both sides with fine netting. For the FSEL#32 strain two cages were used; one was kept with 0.1gr/L tetracycline added in water (FSEL#32/Tet+) and the second one without tetracycline (FSEL#32/Tet-). Three L of pupae (approx. 170,000 insects in 1:1 sex ratio) were loaded in each cage and provided with 2 kg of adult diet (sugar: hydrolyzed enzymatic yeast as a source of protein-ICN Biomedical; 3:1 by weight) and water *ad libitum*.

The strains were maintained together in the same room (8 m length x 5 m width x 5 m height) and the mass-rearing cages were hung from the ceiling and positioned in a row. The light intensity was 2300 lux at the top, 2860 lux in the middle and 2026 lux at the bottom of the cage. The room was maintained with a 12:12 (L:D) h photoperiod, 23–24°C temperature and 50–60% RH.

Females laid eggs by inserting their ovipositor through the netting and eggs were collected daily during 10 days of oviposition from water poured in metal troughs, placed underneath, on both sides of the cages. Three different egg collection days were selected randomly in each generation and used for the evaluation.

#### Egg incubation

For both sexing strains, the collected eggs were incubated for 48 hours in plastic bottles containing water in an egg:water ratio of 1:20 and “bubbled” with a filtered flow of compressed air to allow egg aeration and suspension.

#### Production lines—VIENNA 8^D53-^ /Toliman GSS

The egg production of VIENNA 8^D53-^/Toliman GSS was split into two batches, one batch was incubated at 23–24°C for 48 h to produce males and females and maintain the colony (colony production line) and the second batch was incubated at 23–24°C for 24 h then switched to 34°C into a water bath for 12 h to kill the female embryos and returned to 23–24°C for the next 12 h to complete the incubation (male-only production line).

#### Production lines–FSEL#32 TSS

The eggs from the cage FSEL#32/Tet+ (colony production line) and the eggs from the cage FSEL#32/Tet- (male-only production line) were incubated at 23–24°C during the whole incubation period.

#### Larval rearing

After incubation, 3 mL of eggs from the colony production lines were seeded on three mass-rearing trays that contained 5 kg of modified wheat bran-based Seibersdorf larval diet [28]. For the male-only production lines, the volume of eggs was doubled in each tray.

### Production efficiency

#### Egg production

The number of eggs produced per female per day was estimated by counting the number of eggs in 0.5 mL and this was multiplied with the total volume of eggs collected daily and divided by the number of females maintained inside each cage.

#### Pupal production

The mature larvae that left the diet were daily collected as pupae in sawdust. The pupae were transported and kept in a dark room (19°C and 50–60% RH) until they completed their development. The volume of produced pupae was measured daily and the number of pupae in a sample of 5 mL was counted to estimate the total number of pupae. The daily volume of pupae was multiplied with the number of pupae per 5 mL to calculate daily pupal production. This allowed an assessment of “egg to pupae” recovery. Five mL of pupae were left in a plastic petri-dish until adult emergence to determine the sex ratio (the pupae of the GSS were separated by colour).

### Quality control indices

Quality control parameters i.e. fertility, pupal weight, flight ability, survival of sterile males under starvation, induced sterility and genetic stability of the strains were assessed following the procedures described in the FAO/IAEA/USDA Manual [29]. The fertility was estimated for each cage in the colony. Pupal weight, and flight ability tests were carried out for both colony and male-only production lines in triplicate for four generations. Survival of sterile males under starvation was assessed for the male-only production line as of the fourth generation. To induce sterility, the pupae were irradiated with 120 Gy in a Co^60^ irradiator (Gamma Cell 220, Nordion, Canada) 24–48 h before male emergence.

#### Fertility

Fertility was assessed as hatch rate from three samples of 100 eggs taken from the egg-collections destined for both the maintenance of production and male-only lines. The eggs from the GSS of the male-only production line were taken immediately after the heat treatment. The eggs were aligned on a strip of wet black filter paper and placed in a plastic petri dish that contained a piece of wet sponge to maintain moist. The eggs were incubated at 24–25°C for 5 days and then the number of unhatched eggs was counted for hatch rate determination.

#### Pupal weight

Two days before adult emergence, three samples of pupae (5 mL pupae in each sample) were taken from each generation and their weight and number of pupae per sample measured. In the samples of the GSS, the same procedure was carried out for brown (male) and white pupae (female) separately.

#### Flight ability test

Pupae already separated by color, in the case of the GSS´s, were placed in a special plexiglass tube as described in the FAO/IAEA/USDA Manual [29] and transferred to a flight ability test cage. All flies that were able to fly from the tubes were aspirated from the cage twice a day. After all flies had emerged, the number of non-emerged pupae was counted. The test included an assessment of parameters such as adult emergence, percentage of partially emerged flies, percentage of deformed flies and percentage of fliers. The two most important parameters for an operational sterile insect release program are adult emergence and percentage of fliers which were shown to be closely correlated in the present study. Therefore, only the percentage of fliers is shown.

#### Survival of males under starvation

Fifty male flies of each sexing strain that emerged from pupae irradiated with 120 Gy were aspirated within 2 h following emergence in a petri dish (150 mm diameter) through an opening of 15 mm in the center of the lid, and, after transferring the males, the opening was closed with a rubber stopper. The flies were kept without food and water. The percentage survival was calculated by counting the number of males that had died after 48 h. The test was replicated ten times with males from 3 different cohorts.

### Genetic stability

Twenty mL of pupae from each sexing strain of both production lines (colony and male-only) were sampled for each generation and the number and sex of emerged adults was recorded.

In the case of the GSS, the presence of recombinants was assessed by separating the brown and white pupae and adults according to their sex. For this sexing strain, the most frequent recombination is type-1 and takes place between the translocated regions [30]. There are two different type-1 recombinations: type-1a occurs in the region between the translocation breakpoint and *wp* allele while type-1b occurs in the region between *wp* and *tsl*. The type-1a produces females emerging from brown pupae (*wp*^+^ *tsl*^+^) and males emerging from white pupae (*wp tsl*). The type-1b produces thermo-sensitive males emerging from brown pupae (*wp*^+^ *tsl*) and thermo-resistant females emerging from white pupae (*wp tsl*^+^) only detected in male-only production line. Both recombination type-1a and type-1b events were estimated.

In the case of the TSS, the occurrence of recombinants was assessed by recording the emergence of females in the male-only lines. Flies were observed under an epifluorescent Leica microscope (Wetzlar, Germany) using GFP2 and TxRed filters.

### Induced sterility

To assess potential differences in induced sterility amongst the tested sexing strains, males were irradiated as pupae (2 days before adult emergence) with 60, 80, 100 and 120 Gy. The untreated control group was not irradiated. Fifty treated males from each irradiation dose were housed with 50 untreated females in separate cages. The eggs produced in each cage were collected and 1000 eggs were sampled and aligned on moist black filter paper (for convenience in counting) and transferred to a carrot powder-based larval diet modified from the standard, wheat bran-based diet [31]. After six days of incubation at 24–25°C, egg hatch was recorded and the filter paper was removed from the diet. Thereafter, the total number of pupae and adults obtained in each treatment was recorded. Five replicates from each treatment were evaluated.

### Mating competitiveness of sterile males in walk-in field cages

Four walk-in field cages (3 m diameter x 2 m height), made of nylon netting that was supported by an octagonal frame of PVC pipes [32, 33] were placed in an insect greenhouse with controlled environment (temperature of 23–24°C and 45–60% R.H.). A potted sweet orange tree *Citrus sinensis* (L.) with a height of 1.7 m was placed in the centre of each field cage.

The mating competitiveness of sterile males was assessed in the walk-in field cages with sterile males from the male-only production line competing with fertile wild males for fertile wild females. The males from sexing strains were sterilized with a radiation dose of 120 Gy administered to pupae 2 days before adult emergence. The sterile males were obtained from the male-only production line, whereas the fertile wild flies were sorted by sex within the first 24 h of emergence. All flies were maintained at 23–24°C and a photoperiod of 12:12 h light:dark (8:00–20:00 h) with adult food (3:1 sugar: hydrolysed yeast) and water *ad libitum*. The males were marked with a non-toxic color dye (Brauns-Heitmann Crazy Colors Lebensmittelfarbe‎, www.brauns-heitmann.de) that was added to the male’s diet and different colors were used to distinguish the different treatments. To avoid any bias the colors were rotated among treatments and replicates. The wild females were transferred to another room having the same environmental conditions to prevent their exposure to the male sex pheromones.

Once the insects reached sexual maturity (4–7 days post-emergence), 50 males of each sexing strain and 50 wild males were released in each cage at 08:30 h. Thirty minutes after releasing the male flies, 50 wild females were released in the cages. As soon as the flies started mating, the individual couples were collected in small glass vials. Collection of the mating pairs was stopped when the males ceased their sexual calling activities, at around 12:00 h. All mating pairs collected were frozen and observed under a stereomicroscope to determine the identity of the males by observing the color in the gut. Three different cohorts of males were analysed. Four replicates were performed in the first cohort and three in the next ones in order to obtain a total set of 10 replicates.

### Data analysis

The parameters expressed in percentage were ArcSine√x+1 transformed [34] for all assessments to achieve normal distribution.

#### Production and quality control

A repeated measures multivariate ANOVA was used for the comparison of daily egg production between the three cages of the production lines (VIENNA 8^D53-^/Toliman GSS, FSEL#32/Tet+ and FSEL#32/Tet-). Additionally, one-way ANOVA was carried out for the egg production of each oviposition day and Tukey-Kramer HSD test was used for post hoc comparison of the oviposition days that were significantly different from each other.

Student’s *t*-test was used for the comparison of other parameters (fertility, egg to pupae conversion, weight of pupae, fliers and male starvation survival) between sexing strains (GSS and TSS) for each production line.

#### Estimation of male pupae production cost

Biological, production and quality control parameters, the cost of diet ingredients and supplies involved in the production process as well as the equipment cost and depreciation were entered in the FAO/IAEA Interactive Spreadsheet for Designing and Operating Insects Mass-rearing Facilities (SDIMF) [35]. The rearing costs per million of male pupae (US$) was estimated for each of the twelve replicates of pupae produced during the experiment at different production levels (100, 500, 1000 and 2500 million of pupae per week) for each sexing strain. The estimated costs include both production lines and the cost of FRS, which is an essential component to maintain the integrity of the sexing system in both strains. The pupae (male-only) production cost of the sexing strains was compared in each production level by an Student’s *t*-test while one-way analysis of variance (ANOVA) was used for comparing the cost between the different production levels.

#### Sterility

Each parameter (egg hatch, pupae and adult survival) of the sexing strains was compared in each radiation dose by an Student’s *t*-test, including the fertility of untreated males.

#### Male sexual performance

Prior to the analysis of sexual competitiveness, the proportion of mating (PM = number of mating pairs collected/number of females released) was assessed for each field cage (replicate) to measure the suitability of the flies and the environment for mating; only with a PM > 0.2 can the test be considered valid for an analysis [29]. A chi-square analysis using a likelihood ratio test on the total number of matings recorded from ten replicates was used to determine significant differences in mating between males of the two strains (sexing strain *vs* wild population). In addition, the relative sterility index (RSI = Number of matings of sterile males of the sexing strain/Total number of matings of wild females) was used to estimate the sexual competitiveness of each sexing strain. The RSI values range between 0 and 1 and values close to 0.50 indicate equal sexual performance of the two types of male [36]. The RSI values were compared using Student’s *t* test.

All analyses were performed using the JMP v.7 (SAS institute, Cary, NC, USA) software.

## Results

### Production efficiency

#### Egg production

**Fig 1:**
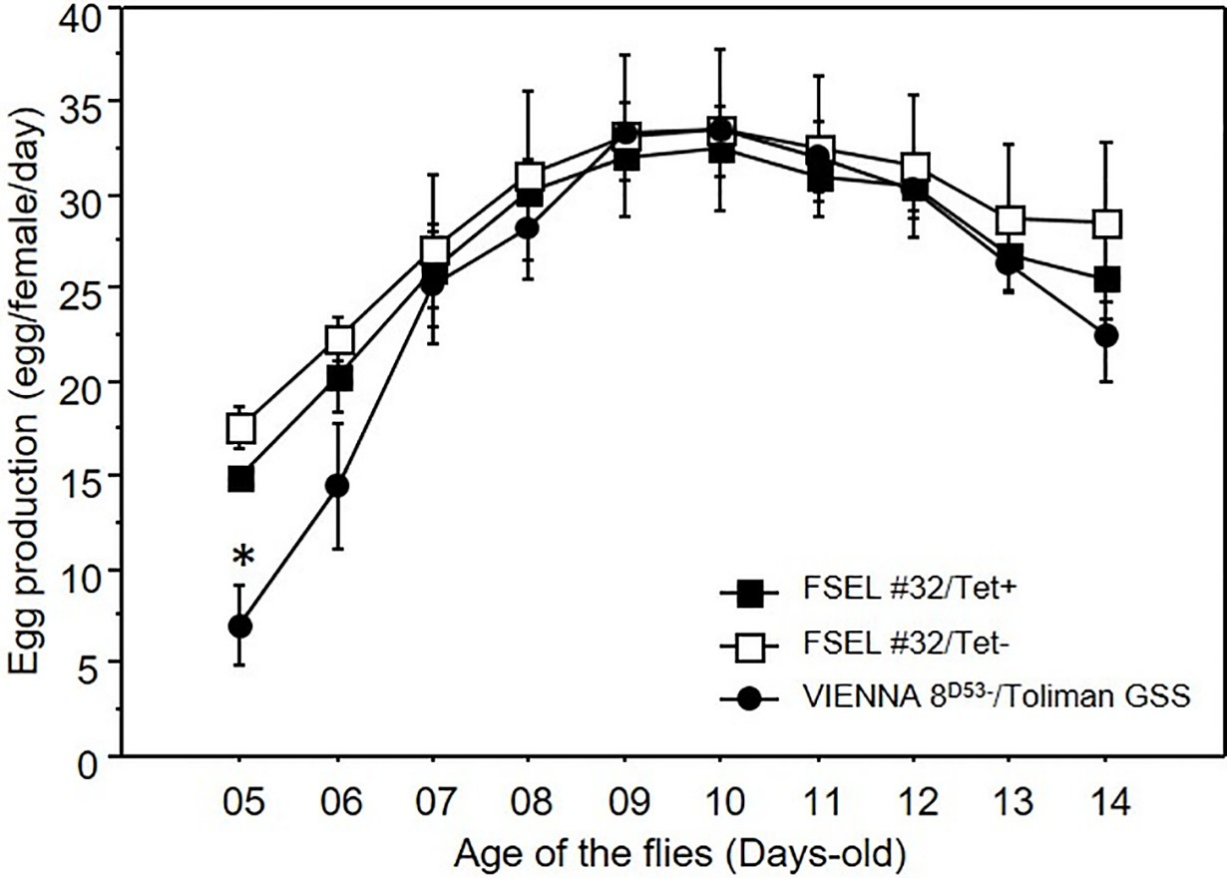
Average eggs production profile (Mean ± SE) under semi-mass rearing conditions of the sexing strains; VIENNA 8^D53-^/Toliman GSS and FSEL#32 with tetracycline (Tet+) or without tetracycline (Tet-) assed during 4 different generations. * = Significant difference.

The females started ovipositing eggs 4 days after emergence. However, very few eggs were produced on the 4^th^ day post emergence and these were excluded from further analysis. Only eggs produced between day 5 and day 15 post emergence were considered for comparisons. For the 4 generations of the assessment, a statistically significant difference was observed among the days of oviposition (*F*_*2*,*9*_ = 37.546, *P* < 0.001). The females oviposited most of the eggs between days 9–14 post emergence, after which egg production declined slightly and no significant difference was detected in the interaction sexing strain per days of oviposition (*F*_*2*,*9*_ = 0.963, *P* > 0.05). Only on the 1st day, the GSS produced fewer eggs per female than the two TSS lines (*F*_*2*,*9*_ = 15.353, *P* < 0.05) (Fig 1).

The GSS and the two TSS colonies produced similar number of eggs for the entire experimental period (*F*_*2*,*117*_ = 157.174, *P* > 0.05) (Table 1).

**Table 1:** Productivity and quality control parameters of the colony production and male- only production lines of different genetic sexing strains of *Ceratitis capitata*.

Parameters	Colony production line		Male-only production line	
	VIENNA 8 ^D53-^/Toliman GSS	FSEL #32/Tet+ TSS	VIENNA 8 ^D53-^/Toliman GSS	FSEL #32/Tet- TSS
Egg production (egg/female/day)	25.30 ± 1.45 a	26.98 ± 0.97 a	NA	28.62 ± 1.29 a
Fertility (%)	76.45 ± 0.63 a	77.55 ± 0.70 a	49.29 ± 0.63 b	53.66 ± 0.76 a
Eggs to pupae conversion (%)	59.69 ± 2.33 b	66.78 ± 1.63 a	30.82 ± 0.51 b	34.64 ± 1.37 a
Weight brown pupa (mg)	8.66 ± 0.06 aA	^&^8.41 ± 0.08 b	8.61 ± 0.05 a	8.04 ± 0.04 b
Weight white pupa (mg)	9.43 ± 0.15 aB	NA	NA	NA
Fliers (%)	88.47 ± 0.76 aA	^&^88.29 ± 0.70 a	84.92 ± 1.40 a	87.93 ± 0.41 a
Female fliers (%)	78.58 ± 2.18 aB	NA	NA	NA
Male starvation survival (%)	ND	ND	80.80 ± 0.61 a	61.40 ± 1.40 b
Relative Sterility Index (RSI)	ND	ND		0.40 ± 0.01 a	0.41 ± 0.02 a

Mean ± standard error followed by the same letter are not statistically different from each other (*P* < 0.05).

Lower-case letters = analysis between sexing strains; upper-case letters = analysis between parameters; NA = not applied; ND = not determined

^&^ male and female.

#### Pupal production

**Fig 2:**
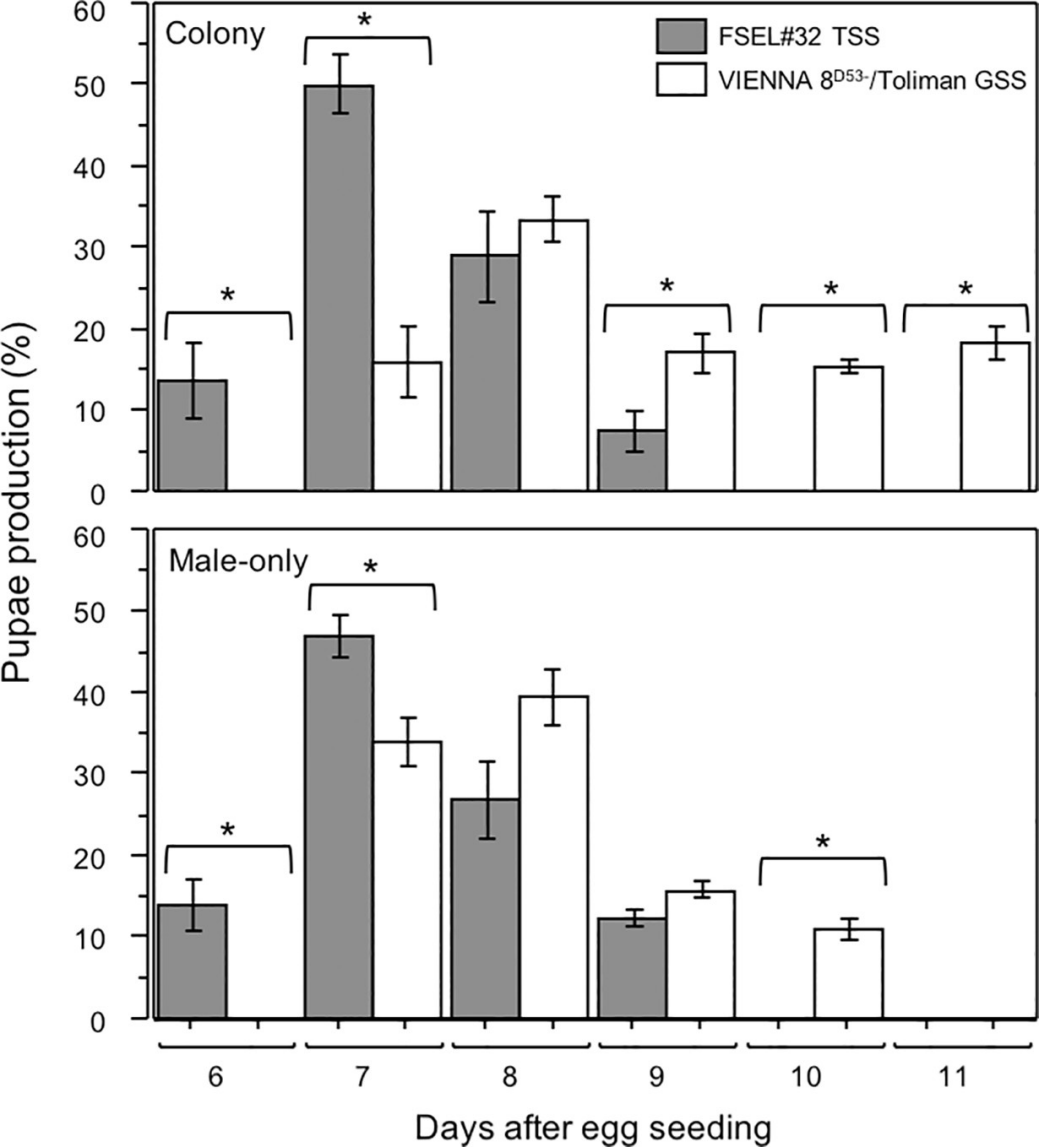
Pupae production profile (Mean ± SE) under semi mass-rearing conditions of the genetic sexing strain VIENNA 8^D53-^/Toliman, transgenic sexing strain FSEL#32 for both production lines. * = Significant difference (P < 0.05).

The pupal production was significantly different between sexing strains during the whole production period with only the following exceptions: the 8^th^ day in the colony production line (*t* = 0.93, *df* = 66, *P* > 0.05) and the 8^th^ (*t* = 1. 93, *df* = 6, *P* > 0.05) and 9^th^ day (*t* = 2.23, *df* = 6, *P* > 0.05) in the male-only production line. The TSS started to produce pupae one day earlier than the GSS, i.e. on day 6 after the seeding of the eggs and all pupae were produced in 4 days for both production lines. Pupae production in the GSS lasted for 5 days in the colony production line and 4 days in the male-only production line. For the TSS, the pupae production spiked on the 7^th^ day after seeding, while this was one day later for the GSS (Fig 2).

The sex ratio of the offspring of the different sexing strains was not significantly different (*t* = 2.33, *df* = 6, *P* > 0.05). However, whereas a 1:1 sex ratio was observed during each pupae collection day for the FSEL#32/Tet+ TSS strain, no white pupae (females) were obtained on the 1st day of pupae collection for the VIENNA 8^D53-^/Toliman GSS, and 10%, 39%, 85% and 93% of white pupae were collected on successive days, respectively.

Overall, the egg to pupae conversion of the TSS was significantly higher than that of the GSS in both the colony (*t* = -2.57, *df* = 22, *P* < 0.05) and the male-only production line (*t* = -2.63, *df* = 22, *P* < 0.05) (Table 1).

### Quality control indices

#### Fertility

Fertility (% egg hatch) was similar in the colony production line of both sexing strains (*t* = -1.00, *df* = 70, *P* > 0.05). However, in the male-only production line, the fertility was significantly higher for the TSS than for the GSS (*t* = -4.55, *df* = 70, *P* < 0.05) (Table 1).

#### Pupal weight

The weight of the brown male pupae for the GSS was significantly higher than that of the TSS in both production lines (colony: *t =* 2.67, *df* = 24, *P* < 0.05; male-only: *t =* 8.07, *df* = 22, *P* < 0.05).

An additional analysis, comparing the weight of brown pupae (males) and white pupae (females) of the VIENNA 8^D53-^/Toliman GSS, showed that the weight of the male pupae was significantly lower than the weight of the female pupae (*t* = 5.03, *df* = 24, *P* < 0.05) (Table 1).

#### Flight ability test

No significant difference was detected in the percentage of male fliers of the sexing strains in both production lines (colony: *t* = 0.16, *df* = 22, *P* > 0.05; male-only: *t* = -2.06, *df* = 22, *P* > 0.05) and for the GSS, the percentage of fliers was significantly higher for males than for females (*t* = 4.23, *df* = 22, *P* < 0.05) (Table 1).

#### Survival of males under starvation

Males of the GSS survived significantly longer than TSS males under starvation (*t* = -11.45, *df* = 18, *P* < 0.05) (Table 1).

### Genetic stability

Both production lines of the GSS (colony and male-only) showed significant accumulation of type-1a recombinants, particularly females emerging from brown pupae (*wp*^+^), under semi-mass rearing for four generations. However, there was no significant accumulation of *wp* males. Type-1b recombination also occurred in the GSS male-only production line and was characterized by the accumulation of *wp* females in the range of 0.66% to 3.48%. The genetic stability of the TSS was assessed through the presence of females in the male-only production line, and only a single female was detected in the 4^th^ generation. This female fly showed only green fluorescence under a fluorescent microscope suggesting the absence of the driver resulting in an incomplete transgenic sexing system (Table 2).

**Table 2:** Stability of the classical genetic sexing (VIENNA 8^D53-^/Toliman) and the transgenic sexing (FSEL#32) strains of *Ceratitis capitata*, under semi mass rearing conditions.

Production line	Strains	Gen.	Adults emerging from					Recombinant (%)			
			brown pupae		white pupae		Total	Type 1a		Type 1b	
			male	female	male	female		WT female	*wp* male	WT male	*wp* female
Colony	VIENNA 8^D53-^/Toliman GSS	1	616	1	0	417	1034	0.10	0.00	ND	ND
		2	664	3	0	446	1113	0.27	0.00	ND	ND
		3	589	15	2	382	988	1.52	0.20	ND	ND
		4	578	22	2	384	986	2.23	0.20	ND	ND
Male-only	VIENNA 8^D53-^/Toliman GSS	1	897	1	0	6	904	0.11	DHS	DHS	0.66
		2	884	2	0	10	896	0.22	DHS	DHS	1.12
		3	912	10	0	25	947	1.06	DHS	DHS	2.64
		4	918	24	0	34	976	2.46	DHS	DHS	3.48
	FSEL#32/Tet- TSS	1	1058	0	NA	NA	1059	NA	NA	NA	NA
		2	1039	0	NA	NA	1039	NA	NA	NA	NA
		3	1028	0	NA	NA	1028	NA	NA	NA	NA
		4	1071	1 ^G^	NA	NA	1071	NA	NA	NA	NA

NA = Not Apply; ND = Not Detected; DHS = Dead with the heat shock

^G^ Green fluorescence

### Induced sterility test

Male fertility in both GSS and TSS decreased significantly with increasing irradiation dose. The hatch rate of eggs produced by wild females mated with FSEL#32/Tet- males treated with 60 (*t* = -2.31, *df* = 8, *P* < 0.05), 80 (*t* = -2.28, *df* = 8, *P* < 0.05) and 100 Gy (*t* = -2.33, *df* = 8, *P* < 0.05) was significantly higher than the hatch rate of eggs produced by females mated with GSS males treated with the same irradiation doses. However, no significant difference was observed between the sexing strains in the survival of pupae and adult for the same irradiation doses (60, 80 and 100 Gy). For the group of males irradiated with 120 Gy, which is the irradiation dose used in most SIT operational programs against the Mediterranean fruit fly, no significant difference was observed between the sexing strains for all parameters assessed (Table 3).

**Table 3:** Egg hatch, egg to pupae recovery and egg to adult recovery from irradiated males of different sexing strains of *Ceratitis capitata* (VIENNA 8^D53-^/Toliman GSS and FSEL#32/Tet- TSS).

Doses	Sexing Strain	Egg hatch (%)	Pupae survival (%)	Adult survival (%)
0	GSS	77.31±2.40 a	55.12±3.75 a	50.52±4.48 a
	TSS	78.84±2.69 a	58.32±3.05 a	52.48±3.38 a
60	GSS	3.15±0.90 b	1.92±0.27 a	1.76±0.23 a
	TSS	3.70±0.24 a	3.08±0.54 a	2.50±0.38 a
80	GSS	1.44±0.10 b	1.04±0.15 a	0.84±0.18 a
	TSS	2.38±0.39 a	1.74±0.30 a	1.16±0.22 a
100	GSS	0.84±0.08 b	0.58±0.10 a	0.46±0.10 a
	TSS	1.36±0.18 a	1.04±0.15 a	0.76±0.22 a
120	GSS	0.60±0.13 a	0.28±0.15 a	0.16±0.09 a
	TSS	0.52±0.11 a	0.32±0.10 a	0.24±0.07 a

Mean ± standard error followed by the same letter between *Ceratitis capitata* strains within each irradiation regime are not statistically different from each other (P < 0.05).

### Mating competitiveness under field cage conditions

The overall mating success into the cages was very high (more than 80%) for both GSS and TSS reflecting the suitability of the flies and of the field cage environment (FSEL#32/Tet- TSS = 0.88±0.02 and VIENNA 8^D53-^/Toliman GSS = 0.86±0.03).

The males of the wild population achieved significantly more matings than the sterile males of both sexing strains; FSEL#32/Tet- *vs* Wild (*x*^*2*^ = 24.95, *df* = 1; *P* < 0.05), VIENNA 8^D53-^/Toliman GSS *vs* Wild (*x*^*2*^ = 24.95, *df* = 1; *P* < 0.05). However, no significant difference was detected in the RSI for both sexing strains (Table 1).

### Estimation of production cost

The cost of producing one million sterile pupae at a production level of 100 million sterile pupae per week, was USD 348.7±2.5 for the VIENNA 8^D53-^/Toliman GSS and USD 342.1±2.8 for the FSEL#32 TSS, but this difference was not significant (*t* = -1.76, *df* = 22, *P* < 0.05). The cost of producing 1 million pupae decreased significantly when the production levels increased to 500, 1000 and 2500 million pupae per week, i.e. for the GSS from USD 348.7±2.5/1 million for a 100 million production level to USD 155.4±2.4/1 million for a 2500 million production level (*F*_3, 44_ = 1511.09, *P* < 0.05) and for the TSS from USD 342.1±2.8/1 million for a 100 million production level to USD 149.1±3.7/1 million for a 2500 million production level (*F*_3, 44_ = 665.60, *P* < 0.05).

## Discussion

The present study focused on the comparative evaluation of two Mediterranean fruit fly genetic sexing strains, the classical VIENNA 8^D53-^/Toliman GSS and the FSEL#32 TSS, with respect to their production efficiency, standard quality control indices, genetic stability under semi-mass rearing conditions, the effect of irradiation on male mating competitiveness in walk-in field cages, as well as the production cost. The results can be summarized as follows: (a) both sexing strains showed similar egg production; however, the egg to pupae conversion was better for the FSEL#32 TSS; (b) both sexing strains showed similar levels of fertility (egg hatch) and male flight ability; however, the VIENNA 8^D53-^/Toliman GSS produced heavier pupae and the GSS males showed higher survival rates under starvation than FSEL#32 TSS males; (c) the sterile males from both sexing strains showed similar mating competitiveness, and (d) the cost of producing males was similar for both the transgenic FSEL#32 TSS and the VIENNA 8^D53-^/Toliman GSS.

Due to increased cost-effectiveness, currently all area-wide integrated pest management (AW-IPM) programmes incorporating the SIT against Mediterranean fruit fly are using GSSs, either VIENNA 7 or 8 (this last one, with or without inversion) [20, 37, 38, 39]. Despite the genetic instability observed in the earlier GSS, the currently used GSS that are based on male-linked translocations have been very efficient, stable and consistent when protocols for their utilization under mass rearing conditions are implemented properly. The breakdown of genetic sexing systems, which was the main weakness of the earlier developed GSS, was mitigated by incorporating a FRS in the rearing process, and the inclusion of inversions further increased the genetic stability of these strains [4, 13, 21]. Transgenic approaches have been used as an alternative to address some of the limitations of the classical GSSs, such as the long periods required for their development [40, 41, 42]. Evaluation of TSSs of the Mediterranean fruit fly developed so far under a semi mass-rearing environment showed that some of these strains were inferior with respect to certain quality and biological parameters in comparison with the classical GSS [43, 44]. In the current study, the FSEL#32 TSS showed good stability under semi mass-rearing conditions for 4 generations; however, its long-term stability would still need to be evaluated under large-scale mass-rearing conditions (i.e. >2000 million per week) [45, 46, 47]. Therefore, the inclusion of a FRS using the selectable fluorescent markers (green and / or red) would be a prerequisite, and thus may eliminate any potential advantage of using the TSS over the GSS.

One of the most critical parameters for the successful implementation of the SIT is the capability of sterile males to transfer their sterile sperm to wild females. This is only possible when the sterile males can compete successfully with wild males for mating with wild females. In the tests conducted under semi natural field cage conditions, the mating competitiveness of the sterile males of both sexing strains was lower than that of the wild males. However, the RSI value of both strains in comparison with wild males was around 0.4, which is quite good and close to the indices of equal mating i.e. 0.5. The unequal mating success between sterile and wild males is not uncommon and has been attributed to the effects of colonization, mass-rearing under artificial holding conditions [48] and irradiation [49, 50]. Although the negative effects due to the mass-rearing environment are unavoidable, mitigating measures can be undertaken to restore some of the lost quality by “colony refreshment” [51], treating the flies destined for release with chemicals [ginger root oil (GRO) in the case of the Mediterranean fruit fly] to enhance their mating performance [52] and manipulating their microbiota [53]. In addition, GSS based on translocations are inherently semi-sterile and would in principle require a lower irradiation dose to confer adequate sterility in the males as was demonstrated with the ‘comby-fly’ constructs [54, 55]. The current study also showed that VIENNA 8^D53-^/Toliman GSS males irradiated with 60, 80 and 100 Gy induced after mating a higher level of sterility in wild females as compared to males from the FSEL#32 TSS treated with the same doses. However, in operational AW-IPM programmes against the Mediterranean fruit fly that have an SIT component, a higher dose (120 Gy) is being applied to ensure complete sterility of males. At that dose, no significant difference in the egg hatch or adult viability was observed between the two sexing strains.

Cost is always an important factor when producing large numbers of insects but reducing the production costs should never compromise the biological quality traits of the produced sterile males. The rearing cost of one million sterile pupae of the classical Mediterranean fruit fly GSS has been estimated at USD 250–500, depending on the location of the rearing facility and the production level [56, 57]. The Mediterranean fruit fly mass rearing facility “El Pino” located in Guatemala has a capacity to produce more than 2500 million sterile VIENNA 8^D53-^ GSS males per week and the cost of producing one million sterile males varied from USD 378, USD 216 and USD 155 for pupal production levels of 300, 1600 and 2500 million, respectively [58]. Although there was no statistically significant difference, the cost of producing sterile males of the FSEL#32 TSS was slightly lower which may result in considerable savings on a yearly basis. However, caution is needed in drawing a firm conclusion at this stage in view that the tests of the present study were done under semi mass-rearing conditions and should be repeated under real large-scale levels. In addition, the lower average weight of the FSEL#32 TSS pupae might be a direct consequence of the high larval survival resulting in high larval density, which indirectly reduced adult survival under stress conditions. The lower adult survival could in addition have been affected by the intake of tetracycline that affects the microbiome. The decrement of both parameters should not be neglected as these are both important quality control parameters.

Taken together, safe realistic conclusions on the potential use of FSEL#32 TSS can only be drawn after the evaluation of this strain under large scale mass-rearing and open field conditions to complete the evaluation phases to which any new strain, developed by classical genetic or transgenic based technologies, have to be submitted (Table 4) [59]. In addition, a risk assessment analysis would be required to address the concerns related to the potential release of transgenic strains in ecosystems as well as to obtain the necessary regulatory approvals for open field releases [60, 61].

**Table 4:** Evaluation phases for the classical VIENNA 8^D53-^/Toliman GSS and the transgenic FSEL#32/Tet-: from the laboratory to operational programs.

Required	Sexing Strain	
Rearing scale	VIENNA 8^D53-^/Toliman GSS	FSEL#32/Tet-TSS
Laboratory	Done	Done
Semi mass-rearing	Done	Done
Mass-rearing	Done	Pending
**Genetic sexing function**		
Permissible conditions	Done	Done
Restrictive conditions	Done	Done
**Productivity**		
Pre-oviposition period	Done	Done
Oviposition profile	Done	Done
Fecundity	Done	Done
Colony larval development time	Done	Done
Male larval development time	Done	Done
Pupal development time	Done	Done
Egg to pupae efficiency	Done	Done
Cost/million pupae	Done	Done
**Quality control of product**		
Pupal weight	Done	Done
Flight ability	Done	Done
Adult longevity under stress	Done	Done
Sterility test	Done	Done
**Field cage performance**		
Sexual competitiveness	Done	Done
Sterility index	Done	Pending
**Open field**		
Longevity	Done	Pending
Dispersal	Done	Pending
Marker persistence	Done	Pending
Response to traps and lures	Done	Pending

## References

[1] White IM, Elson-Harris MM. Fruit Flies of Economic Significance: Their Identification and Bionomics. London, UK: CAB International; 1992.

[2] Liquido NJ, Shinoba LA, Cunningham RT. Host plants of the Mediterranean fruit fly (Diptera: Tephritidae): an annotated world review. Misc Publ Entomol Soc Am. 1991; 77: 1–52.

[3] Rossler Y. Insecticidal bait and cover sprays In: Robinson AS, Hooper G, editors. Fruit Flies, Their Biology, Natural Enemies and Control, World Crop Pests 3B. Amsterdam, The Netherlands: Elsevier; 1989 p. 329–336.

[4] Vreysen MJ, Hendrichs J, Enkerlin WR. The sterile insect technique as a component of sustainable area-wide integrated pest management of selected horticultural insect pests. J Fruit Ornam Plant Res. 2006; 14: 107.

[5] Enkerlin W, Gutiérrez-Ruelas JM, Cortes AV, Roldan EC, Midgarden D, Lira E, et al Area freedom in Mexico from Mediterranean fruit fly (Diptera: Tephritidae): a review of over 30 years of a successful containment program using an integrated area-wide SIT approach. Fla Entomol. 2015; 98: 665–681.

[6] Knipling EF. Possibilities of insect control or eradication through the use of sexually sterile males. J Econ Entomol. 1955; 48: 459–462.

[7] Dyck VA, Flores JR, Vreysen MJ, Fernandez ER, Teruya T, Barnes B, et al Management of Area-Wide Integrated Pest Management Programmes that Integrate the Sterile Insect Technique In: Dyck VA, Hendrichs J, Robinson AS, editors. Sterile Insect Technique Principles and Practice in Area-Wide Integrated Pest Management. Dordrecht, The Netherlands: Springer; 2005 p. 427–451.

[8] Cáceres C. Mass rearing of temperature sensitive genetic sexing strains in the Mediterranean fruit fly (*Ceratitis capitata*). Genetica. 2002; 116: 107–116. 12484530 10.1023/a:1020967810703

[9] Parker AG. Mass-rearing for sterile insect release In: Dyck VA, Hendrichs J, Robinson AS. editors. Sterile Insect Technique Principles and Practice in Area-Wide Integrated Pest Management. Dordrecht, The Netherlands: Springer; 2005 p. 209–232.

[10] Robinson AS, Van Heemert C. *Ceratitis capitata*—a suitable case for genetic sexing. Genetica. 1982; 58: 229–237.

[11] Ozaki ET, Kobayashi RM. Effects of duration and intensity of sifting pupae of various ages on adult eclosion and flight capability of the Mediterranean fruit fly (Diptera: Tephritidae). J Econ Entomol. 1982; 75: 773–776.

[12] Franz G, Gencheva E, Kerremans P. Improved stability of genetic sex-separation strains for the Mediterranean fruit fly, *Ceratitis capitata*. Genome. 1994; 37: 72–82. 18470062 10.1139/g94-009

[13] Franz G. 2005 Genetic sexing strains in Mediterranean fruit fly, an example for other species amenable to large-scale rearing as required for the sterile insect technique In: Dyck VA, Hendrichs J, Robinson ASeditors. Sterile Insect Technique Principles and Practice in Area-Wide Integrated Pest Management. Dordrecht, The Netherlands: Springer; 2005 p. 427–451.

[14] Cáceres C, Cayol JP, Enkerlin WR, Franz G, Hendrichs J, Robinson AS. Comparison of Mediterranean fruit fly (*Ceratitis capitata*) (Tephritidae) bisexual and genetic sexing strains: development, evaluation and economics In: Barnes BN, editor, Proceedings, Symposium: 6^th^ International Symposium on Fruit Flies of Economic Importance, 6–10 May 2002, Stellenbosch, South Africa. Isteg Scientific Publications, Irene, South Africa; 2004 p. 367–381.

[15] Hendrichs J, Franz G, Rendon P. Increased effectiveness and applicability of the sterile insect technique through male‐only releases for control of Mediterranean fruit flies during fruiting seasons. J Appl Entomol. 1995; 119: 371–377.

[16] Rendón P, McInnis D, Lance D, Stewart J. Medfly (Diptera: Tephritidae) genetic sexing: large-scale field comparison of males-only and bisexual sterile fly releases in Guatemala. J Econ Entomol. 2004; 97:1547–1553. 15568342 10.1603/0022-0493-97.5.1547

[17] Kerremans P, Bourtzis K, Zacharopoulou A. Cytogenetic analysis of three genetic sexing strains of *Ceratitits capitata*. Theor Appl Genet. 1990; 80: 177–182. 10.1007/BF00224383 24220892 10.1007/BF00224383

[18] Kerremans P, Franz G. Cytogenetic analysis of chromosome 5 from the Mediterranean fruit fly, *Ceratitis capitata*. Chromosoma. 1994; 103: 142–146. 8055711 10.1007/BF00352323

[19] Kerremans P, Franz G. Isolation and cytogenetic analyses of genetic sexing strains for the medfly, *Ceratitis capitata*. Theor Appl Genet. 1995; 91: 255–261. 10.1007/BF00220886 24169772 10.1007/BF00220886

[20] Augustinos AA, Targovska A, Cancio-Martinez E, Schorn E, Franz G, Cáceres C, et al *Ceratitis capitata* genetic sexing strains: laboratory evaluation of strains from mass rearing facilities worldwide. Entomol Exp Appl. 2017 164; 305–317.

[21] Fisher K, Cáceres C. A filter rearing system for mass reared genetic sexing strains of Mediterranean fruit fly (Diptera: Tephritidae) In: Tan KH. Editor Area-wide Control of Fruit Flies and Other Insect Pests. Penang, Malaysia: Penerbit Universiti Sains Malaysia 2000 p. 543–550.

[22] Scolari F, Schetelig MF, Gabrieli P, Siciliano P, Gomulski LM, Karam N, et al Insect transgenesis applied to tephritid pest control. J Appl Entomol. 2008; 132: 820–831.

[23] Gong P, Epton MJ, Fu G, Scaife S, Hiscox A, Condon KC. A dominant lethal genetic system for autocidal control of the Mediterranean fruit fly. Nat Biotechnol. 2005; 23: 453–456. 10.1038/nbt1071 15750586 10.1038/nbt1071

[24] Morrison NI, Franz G, Koukidou M, Miller TA, Saccone G, Alphey L, et al Genetic improvements to the sterile insect technique for agricultural pests. Asia Pac J Mol Biol Biotechnol. 2010; 18:275–295.

[25] Fu G, Condon KC, Epton MJ, Gong P, Jin L, Condon GC, et al Female-specific insect lethality engineered using alternative splicing. Nat Biotechnol. 2007; 25: 353–357. 10.1038/nbt1283 17322873 10.1038/nbt1283

[26] Schetelig MF, Cáceres C, Zacharopoulou A, Franz G, Wimmer EA. Conditional embryonic lethality to improve the sterile insect technique in *Ceratitis capitata* (Diptera: Tephritidae). BMC biology. 2009; 7: 4 10.1186/1741-7007-7-4 19173707 10.1186/1741-7007-7-4 PMC2662800

[27] Ogaugwu CE, Schetelig MF, Wimmer EA. Transgenic sexing system for *Ceratitis capitata* (Diptera: Tephritidae) based on female-specific embryonic lethality. Insect Biochem Mol Biol. 2013; 43: 1–8. 10.1016/j.ibmb.2012.10.010 23137881 10.1016/j.ibmb.2012.10.010

[28] Hooper GHS. Application of quality control procedures to large scale rearing of the Mediterranean fruit fly. Entomol Exp Appl. 1987; 44: 161–167.

[29] FAO/IAEA/USDA. Product Quality Control for Sterile Mass-Reared and Released Tephritid Fruit Flies, Version 6.0. Vienna, Austria: International Atomic Energy Agency; 2014. Available: http://www-naweb.iaea.org/nafa/ipc/public/QualityControl.pdf.

[30] Franz G. Recombination between homologous autosomes in medfly (*Ceratitis capitata*) males: type-1 recombination and the implications for the stability of genetic sexing strains. Genetica. 2002 116; 73–84. 12484527 10.1023/a:1020911725724

[31] Hooper GHS. Application of quality control procedures to large scale rearing of the Mediterranean fruit fly. Entomol Exp Appl. 1987; 44: 161–167.

[32] Chambers DL, Calkins CO, Boller EF, Itô Y, Cunningham RT. Measuring, monitoring and improving the quality of mass‐reared Mediterranean fruit flies, *Ceratitis capitata* (Wied.). J Appl. Entomol. 1983; 95: 285–303.

[33] Calkins CO, Webb JC. A cage and support framework for behavioral tests of fruit flies in the field. Florida Entomol. 1983; 66: 512–514.

[34] Zar JH. Biostatistical analysis, 4th ed Hall Prentice, Englewood Cliffs NJ. 1999.

[35] Cáceres C, Rendón P, Jessup A. The FAO/IAEA spreadsheet for designing and operating insect mass-rearing facilities: Procedures manual. Food and Agriculture Organization of the United Nations. 2012.

[36] McInnis DO, Lance DR, Jackson CG. Behavioral resistance to the sterile insect technique by Mediterranean fruit fly (Diptera: Tephritidae) in Hawaii. Ann Entomol Soc America. 1996; 89: 739–744.

[37] Hendrichs J, Franz G, Rendon P. Increased effectiveness and applicability of the sterile insect technique through male-only releases for control of Mediterranean fruit flies during fruiting seasons. J Appl Entomol. 1995; 119: 371–377.

[38] Rendón P, McInnis D, Lance D, Stewart J. Medfly (Diptera: Tephritidae) genetic sexing: large-scale field comparison of males-only and bisexual sterile fly releases in Guatemala. J Econo Entomol. 2004; 97: 1547–1553. 10.1603/0022-0493-97.5.1547 15568342

[39] Robinson AS, Franz G, Fisher K. Genetic sexing strains in the medfly, *Ceratitis capitata*: development, mass rearing and field application. Trends Entomol. 1999; 2: 81–104.

[40] Franz G, Robinson A. Molecular technologies to improve the effectiveness of the sterile insect technique. Genetica. 2011; 139: 1–5. 10.1007/s10709-010-9543-z 21258957 10.1007/s10709-010-9543-z

[41] Handler AM. Prospects for using genetic transformation for improved SIT and new biocontrol methods. Genetica. 2002; 116: 137–149. 12484533 10.1023/a:1020924028450

[42] Bourtzis K, Hendrichs J. Preface: Development and evaluation of improved strains of insect pests for sterile insect technique (SIT) applications. BMC Genetics. 2014; 15(Suppl 2):I1 25472848 10.1186/1471-2156-15-S2-I1 PMC4255763

[43] Ramírez-Santos EM, Rendón P, Ruiz-Montoya L, Toledo J, Liedo P. Performance of a genetically modified strain of the Mediterranean fruit fly (Diptera: Tephritidae) for area-wide integrated pest management with the sterile insect technique. J Econ Entomol. 2016; 110:24–34. 10.1093/jee/tow239 28011689

[44] Rempoulakis P, Taret G, Haq I, Wornayporn V, Ahmad S, Tomas US, et al Evaluation of quality production parameters and mating behavior of novel genetic sexing strains of the Mediterranean fruit fly *Ceratitis capitata* (Wiedemann)(Diptera: Tephritidae). PloS One. 2016; 11: e0157679 10.1371/journal.pone.0157679 27336737 10.1371/journal.pone.0157679 PMC4918918

[45] Fraser MJ. Insect transgenesis: current applications and future prospects. Annu Rev Entomol. 2012; 57: 267–289. 10.1146/annurev.ento.54.110807.090545 22149266 10.1146/annurev.ento.54.110807.090545

[46] Handler AM. Understanding and improving transgene stability and expression in insects for SIT and conditional lethal release programs. Insect Biochem Mol Biol. 2004; 34: 121–130. 10.1016/j.ibmb.2003.08.005 14871608 10.1016/j.ibmb.2003.08.005

[47] Handler AM. Enhancing the stability and ecological safety of mass‐reared transgenic strains for field release by redundant conditional lethality systems. Insect Sci. 2016; 23: 225–234. 10.1111/1744-7917.12245 26097098 10.1111/1744-7917.12245

[48] Cayol JP. Changes in sexual behavior and in some life history traits of tephritid species caused by mass-rearing processes In: Aluja M, Norrbom A. Editors. Fruit Flies (Tephritidae): Phylogeny and Evolution of Behavior. CRC Press. Boca Raton, FL; 2000 p. 843–860.

[49] Lux SA, Vilardi JC, Liedo P, Gaggl K, Calcagno GE, Munyiri FN, et al Effects of irradiation on the courtship behavior of medfly (Diptera, Tephritidae) mass reared for the sterile insect technique. Florida Entomol. 2002; 85: 102–112.

[50] Barry JD, McInnis DO, Gates D, Morse JG. Effects of irradiation on Mediterranean fruit flies (Diptera: Tephritidae): emergence, survivorship, lure attraction, and mating competition. J Econo Entomol. 2003; 96: 615–622. 10.1603/0022-0493-96.3.615 12852596

[51] Shelly TE. Outcrossing and the mating competitiveness of male Mediterranean fruit flies (Diptera: Tephritidae): results from the world’s oldest mass-reared strain. Proc Hawaian Entomol Soc. 2001; 35: 49–54.

[52] Shelly T, Edu J, Smith E, Hoffman K, War M, Santos R, et al Aromatherapy on a large scale: exposing entire adult holding rooms to ginger root oil increases the mating competitiveness of sterile males of the Mediterranean fruit fly in field cage trials. Entomol Exp Appl. 2007; 123: 193–201.

[53] Ami EB, Yuval B, Jurkevitch E. Manipulation of the microbiota of mass-reared Mediterranean fruit flies *Ceratitis capitata* (Diptera: Tephritidae) improves sterile male sexual performance. The ISME journal. 2010; 4: 28–37. 10.1038/ismej.2009.82 19617877 10.1038/ismej.2009.82

[54] Steffens RJ. The combi-fly, a new concept for genetic control of fruit flies. Naturwissenschaften. 1982; 69: 600–601.

[55] Steffens RJ. Combination of radiation—and translocation—induced sterility for genetic control of fruit flies. Entomol Exp Appl. 1983; 33: 253–258.

[56] Mumford JD. Application of benefit/cost analysis to insect pest control using the sterile insect technique In: Dyck VA, Hendrichs J, Robinson AS. Editors. Sterile Insect Technique Principles and Practice in Area-Wide Integrated Pest Management. Dordrecht, The Netherlands: Springer; 2005 p. 481–498.

[57] Dyck VA, Hendrichs J, Robinson AS. Sterile insect technique: principles and practice in area-wide integrated pest management Springer Science and Business Media Press; 2005.

[58] Hendrichs J, Robinson AS, Cayol JP, Enkerlin W. Medfly area-wide sterile insect technique programmes for prevention, suppression or eradication: the importance of mating behavior studies. Florida Entomol. 2002; 85: 1–13.

[59] Cáceres C, Rendon P, McCombs S, Stefan M. Characterization of genetically modified *Ceratitis capitata* Wied. (Diptera: Tephritidae) to enhance sterile insect technique programs. In: Montoya PJ, Díaz-Fleischer F, Flores-Breceda S, Editors. 7th meeting of the working group on fruit flies of western hemisphere; 2008. pp. 105–108.

[60] FAO/IAEA. Status and risk assessment of the use of transgenic arthropods in plant protection. FAO/IAEA technical meeting, 8–12 April 2002; 2006. IAEA-TECDOC-1483. Available from: http://www.pub.iaea.org/MTCD/publications/PDF/te_1483_web.pdf. Accessed 14 September 2011.

[61] NAPPO (North American Plant Protection Organization). Guidelines for importation and confined field release of transgenic arthropods in NAPPO member countries; 2007. RSPM 27. Available from: http://www.nappo.org/Standards/Std-e.html. Accessed 14 September 2011.

